# Exploring the role of intestinal microbiota in mitigating acute radiation-induced intestinal injury through high-energy X-ray FLASH radiotherapy via metagenomic analysis

**DOI:** 10.3389/fmicb.2025.1601244

**Published:** 2025-10-17

**Authors:** Huan Du, Xiaofei Hao, Binwei Lin, Yihan Zhu, Yiwei Yang, Mingming Tang, Wei Wu, Decai Wang, Bo Lin, Yuwen Liang, Wenqiang Tang, Haonan Xu, Jie Li, Feng Gao, Xiaobo Du

**Affiliations:** ^1^Mianyang Central Hospital, School of Medicine, University of Electronic Science and Technology of China, Mianyang, China; ^2^NHC Key Laboratory of Nuclear Technology Medical Transformation, Mianyang Central Hospital, School of Medicine, University of Electronic Science and Technology of China, Mianyang, China; ^3^Sichuan Clinical Research Center for Radiation and Therapy, Mianyang Central Hospital, Mianyang, China; ^4^Institute of Applied Electronics, China Academy of Engineering Physics, Mianyang, China; ^5^Clinical Medical School, North Sichuan Medical College, Nanchong, China; ^6^School of National Defense Science and Technology College SWUSST, Southwest University of Science and Technology, Mianyang, China

**Keywords:** X-ray FLASH, FLASH effect, metagenomics, mechanism, gut microbiome

## Abstract

**Objectives:**

This study preliminarily examines the potential correlation between the gut microbiome and the protective effects of FLASH radiotherapy (FLASH-RT) on intestinal tissue using metagenomic analysis.

**Methods:**

Compact single high-energy X-ray source (CHEXs) FLASH-RT was employed for FLASH irradiation, while EBT3 radiochromic film and a fast current transformer were used to measure the absolute dose and the pulsed beam characteristics. Sham radiotherapy (control), FLASH-RT (333 Gy/s), and Conventional dose rate radiotherapy (CONV-RT, 0.07 Gy/s) were performed on whole abdomen of normal C57BL/6J female mice (10 Gy, 12 Gy, 14 Gy). At 72 h post-irradiation, intestinal contents from normal C57BL/6J female mice were collected for metagenomic analysis. The survival status, body weight, and damage to normal tissues were observed.

**Results:**

At 28 days post-whole abdomen irradiation with doses of 12 Gy, the survival rate of the FLASH group was higher than that of the CONV group (*p* < 0.05). Histological analysis of intestinal tissues by H&E staining revealed significantly less acute intestinal damage and inflammation in the FLASH group compared to the CONV group. Further macrobiome analysis using LEfSe indicated that the abundance of beneficial bacteria, including *Weissella*, *Lactobacillus ruminis* and *Lactobacillus taiwanensis* was significantly higher in the FLASH group than in the CONV group. Moreover, compared to the CONV group, the FLASH group exhibited significant upregulation of several signaling pathways, including the glycosaminoglycan degradation, PI3K/Akt and arabinogalactan biosynthesis *Mycobacterium* signaling pathway.

**Conclusion:**

Compared to CONV-RT, high-energy X-ray FLASH irradiation exerts radioprotective effects on normal intestinal tissue. Alterations in the gut microbiota and associated signaling pathways may be linked to the protective effects of FLASH.

## Introduction

Radiotherapy is a primary treatment modality for malignant tumors and remains the most widely used therapeutic approach (Schaue and McBride, 2015). Approximately 40% of long-term survivors benefit from radiotherapy, and around 60–70% of cancer patients receive radiotherapy at various stages of their disease (Baumann et al., 2016; Overgaard and Bartelink, 1995). The radiation dose is a critical factor influencing the effectiveness of tumor treatment. As the dose increases, both the local control rate of the tumor and the patient’s survival rate may improve; however, the risk of side effects to normal tissues within the irradiated field also rises (Melia and Parsons, 2023). Despite its efficacy, one of the significant limitations of radiation therapy is the potential damage it can cause to surrounding healthy tissues, particularly the intestines in abdominal and pelvic treatments (Levy et al., 2020). Acute radiation intestinal injury is a common complication characterized by inflammation, epithelial cell loss, and compromised barrier function, often leading to symptoms such as diarrhea, abdominal pain, and malabsorption (Lu et al., 2023). These effects not only impact patient quality of life but can also necessitate treatment interruptions or dose reductions, potentially compromising therapeutic outcomes (Bao et al., 2019).

FLASH radiotherapy (FLASH-RT) is a revolutionary new technology in tumor radiotherapy that can deliver ultra-high dose rates (≥40 Gy/s) within an extremely short duration (milliseconds to microseconds) (Favaudon et al., 2014). Compared to conventional dose-rate radiotherapy (CONV-RT), FLASH-RT offers two major advantages: an extremely brief treatment time and enhanced protection for normal tissues, while maintaining the same tumor cell-killing efficacy as CONV-RT (Hughes and Parsons, 2020). In summary, FLASH-RT can improve the efficiency of radiotherapy, ensure treatment efficacy, and reduce toxicity to normal tissues (Chow and Ruda, 2024; Srinivasan et al., 2024).

Despite these promising results, the mechanisms underlying the FLASH effect remain incompletely understood (Shiraishi et al., 2024). Research indicates that FLASH-RT may modify cellular and molecular responses to radiation, including oxidative stress, DNA damage repair, and immune responses (Hageman et al., 2022; Ma et al., 2024). However, these effects are intricate and necessitate further investigation. A deeper understanding of these mechanisms could enhance clinical applications and facilitate the broader adoption of FLASH-RT in oncology (Yan et al., 2024).

Electrons, kilovolt low-energy X-rays, and protons have been used in preclinical studies of FLASH-RT, but these radiation types are not widely applied in clinical practice (Bourhis et al., 2019; Favaudon et al., 2014; Montay-Gruel et al., 2019; Montay-Gruel et al., 2018; Ryoo et al., 2016; Simmons et al., 2019; Vozenin et al., 2019). Electrons and low-energy X-rays are typically used to treat superficial tumors, but their limited penetration makes them unsuitable for tumors located deeper within the body (Eling et al., 2019; Rahman et al., 2022). Flash protons can be used to treat deep-seated tumors, but their high construction and operational costs hinder widespread adoption (Montay-Gruel et al., 2022). High-energy X-rays are the most commonly used type of radiation in clinical radiotherapy, as they offer deep penetration, small divergence, low radiation intensity, and are affordable for patients (Nath et al., 1984). However, generating ultra-high dose-rate high-energy X-rays is challenging, limiting further research in this area. This study utilized a compact, clinical-grade single high-energy X-ray source (CHEXs) FLASH-RT device. The device is capable of generating ultra-high dose-rate high-energy X-rays and has been validated to induce FLASH effect (Shan et al., 2023). While murine models remain a cornerstone for preclinical FLASH research, there is a growing body of work utilizing *in vitro* 3D models such as spheroids and organoids, which better mimic tissue structure and tumor microenvironments. Durak-Kozica et al. (2023) applied FLASH proton irradiation to 3D cancer spheroids, demonstrating preserved structural integrity and suggesting promising biological effects. Moreover, PET-based studies by Cesar et al. (2024) and image-guided FLASH discussions by Lang (2024) further exemplify the relevance of in vitro FLASH systems in translational settings. This study preliminarily examines the potential correlation between the gut microbiome and the protective effects of FLASH-RT on intestinal tissue using metagenomic analysis.

## Materials and methods

### Irradiation device and dosimetry

**Figure 1:**
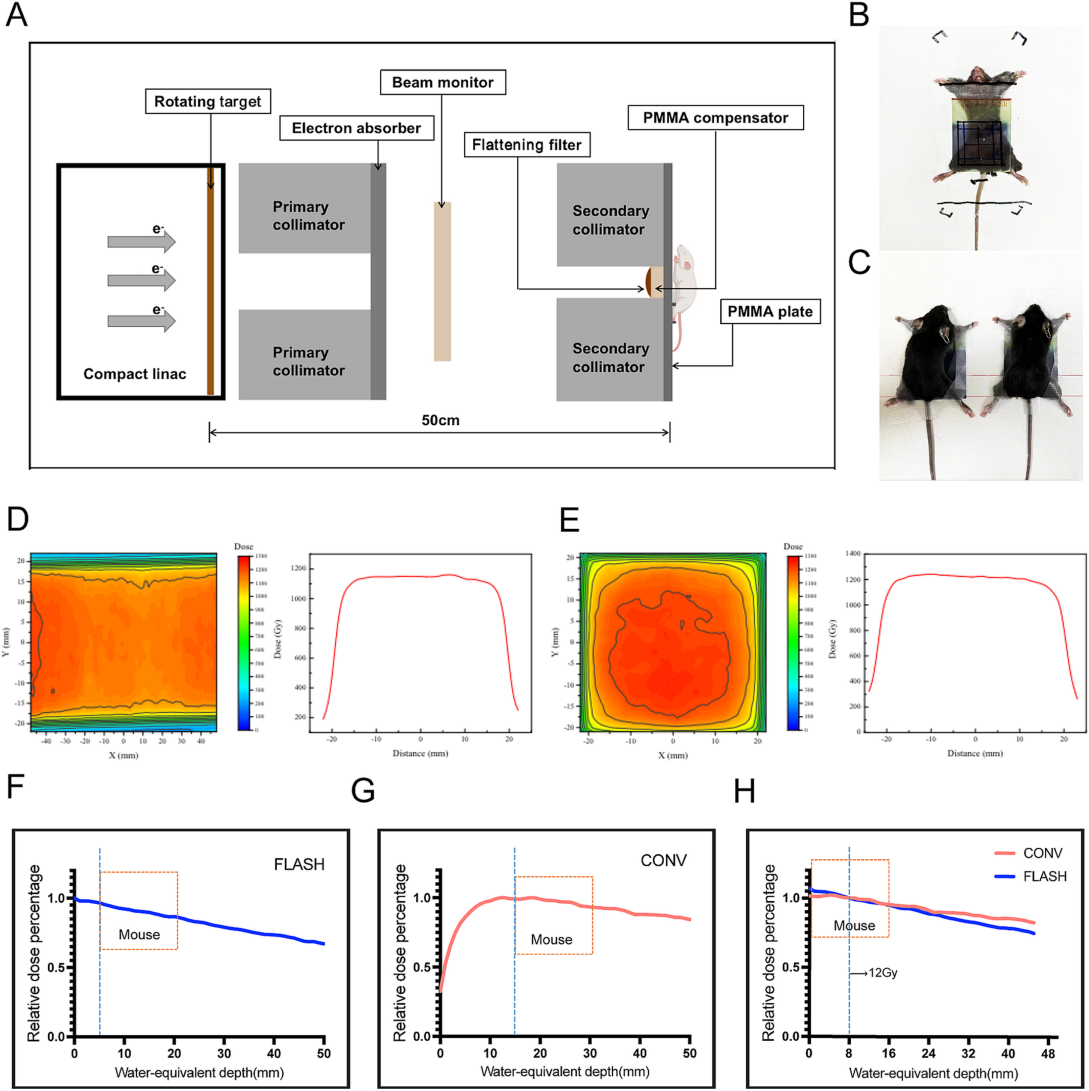
Parameters and dosimetry of FLASH-RT and CONV-RT. **(A)** Schematic diagram of the *in vivo* FLASH-RT experiment. **(B)** For whole-abdomen FLASH-RT, a 5-mm thick polymethyl methacrylate (PMMA) plate was used for dose buildup and mouse fixation. An EBT-XD film was placed between the PMMA plate and the anterior surface of the irradiated mouse to evaluate the dose. **(C)** For whole-abdomen CONV-RT, a 5-mm thick PMMA plate was used for dose buildup and mouse fixation. **(D)** Dose profiles illustrating the dose distribution in FLASH-RT. **(E)** Dose profiles illustrating the dose distribution in CONV-RT. **(F)** PDD curve for X-ray sources used in FLASH-RT, with the mouse placed 5 mm from the secondary collimator and a 5-mm buildup area depth. **(G)** PDD curve for X-ray sources used in CONV-RT, with the mouse placed 15 mm from the secondary collimator and a 15-mm buildup area depth. **(H)** A 12 Gy dose administered 0.8 cm below the surface, showing comparable PDD curves between the FLASH and CONV groups.

FLASH-RT experiments were conducted using CHEXs equipment (Mianyang, China), which delivers an average dose rate of 81.01 Gy/s at a source-to-surface distance (SSD) of 1 meter (Shan et al., 2023). CONV-RT experiments were performed with a clinically applied 6 MV Elekta Precision linear accelerator (Elekta AB, Stockholm, Sweden). Dose monitoring procedures followed previously established protocols (Gao et al., 2022). Beam current was monitored using a brushing-current transformer (BCT), and a diamond detector was positioned downstream of the primary collimator for X-ray beam monitoring. Additionally, Gafchromic^™^ EBT-XD radiochromic films (Ashland Inc., Covington, Kentucky, United States) were placed beneath solid water at the central level of the irradiation target area to ensure uniform dose distribution. Figure 1A illustrates the schematic of the *in vivo* FLASH-RT experiment. Lead secondary collimators with apertures of 4 × 4 cm^2^ was used to define the FLASH irradiation field, and the total irradiation dose was controlled by varying the exposure time. Figures 1B,C shows the setup for fixation and whole-abdomen irradiation of mice in the FLASH-RT and CONV-RT groups. EBT-XD films were placed on the anterior surface of each mouse to monitor the total dose of a single irradiation event (Figures 1D,E). Figures 1F–H presents the percent depth dose (PDD) curves for both FLASH-RT and CONV-RT whole-abdomen irradiations.

### Mice and ethics statement

Female C57BL/6J, aged 6–8 weeks, were procured from Sibeifu Experimental Animal Technology Co., Ltd. (Beijing, China). All experimental mice were purchased in a single batch and underwent a 7-day acclimation period prior to the start of the experiment. During this period, they were fed irradiated, sterilized maintenance chow (Synergic Biotechnology) from the same brand and batch. The diet source remained unchanged throughout the entire experiment. In addition, all mice were housed under consistent environmental conditions, including lighting, temperature, humidity, and cage type. All animal experiments were conducted in accordance with the relevant ethical guidelines and approved by the Animal Ethics Committee of Mianyang Central Hospital (approval number: S20230204).

### Whole abdominal irradiation of normal mice

In the 12 Gy whole abdominal irradiation cohort, each group (FLASH, CONV, and control) included 15 mice, with 10 assigned to survival analysis and 5 sacrificed at 72 h post-irradiation for metagenomic analysis. For the 10 Gy and 14 Gy whole abdominal irradiation cohorts, each group consisted of 10 mice, all of which were used for survival analysis only. The entire abdomen of each mouse was irradiated using a 4 cm (cranial) × 4 cm (lateral) irradiation field, with the upper boundary of the irradiation field located at the lower edges of the lungs (2 cm below the bilateral ear edges). The dose rates for FLASH-RT and CONV-RT were 333 Gy/s and 0.07 Gy/s, respectively. At 72 h post-irradiation, five mice from each group in the 12 Gy cohort were euthanized, and intestinal tissues and contents were collected. The survival status of the remaining mice was monitored; those exhibiting abnormal behavior, such as weight loss exceeding 20% or self-harm, were euthanized.

### H&E staining

Three days after irradiation, mice from the 12 Gy groups were euthanized, and the intestinal tissues were extracted and rinsed with physiological saline. The intestinal lumen was opened using micro scissors, and the intestines were rolled from the posterior end with the lumen facing outward (Ruan et al., 2021). The sample was fixed in formalin overnight, then embedded in paraffin and sliced into 5 microns for H&E staining. A modified Swiss roll-based crypt assay was employed to quantify acute crypt damage induced by ionizing radiation (Groselj et al., 2018). The area with the most severe damage was identified based on two independent assessments, focusing on regions with a depth greater than 3 mm and the fewest crypts. The total number of crypts in areas greater than 3 mm was then calculated for each site. Only crypts with more than 10 cells and no signs of apoptosis were considered as regenerating crypts. The number of remaining crypts per millimeter in each group was calculated.

### Metagenomics

At 72 h post-irradiation, five mice from each group in the 12 Gy cohort were euthanized for intestinal content collection. The intestines were carefully extracted using sterile forceps, with the intestinal contents gently expelled, and then placed into a cryogenic container. Due to insufficient sample volume from one mouse in the CONV group, a total of 12 samples were ultimately included in the metagenomic analysis, with four samples per group (FLASH, CONV, and control). The samples were then rapidly frozen in liquid nitrogen and stored in dry ice for further analysis. The raw paired-end sequences obtained from high-throughput sequencing (Illumina NovaSeq 6000) were first subjected to quality control using Fastp v0.20.1, with low-quality reads (Phred score <20), adapter contamination, and ambiguous bases (N content >10%) removed. An average of 45 million clean reads per sample were retained after filtering. Assembly was conducted using MEGAHIT v1.2.9 with default k-mer sizes, and contigs shorter than 500 bp were discarded. Gene prediction was performed using Prodigal v2.6.3. Taxonomic annotation was based on Kraken2 (v2.1.1) and MMseqs2 (sensitivity = 5.7) with sequence identity threshold set at 97%, and only matches with alignment confidence scores ≥0.9 were retained for downstream analysis. Functional annotation was performed against KEGG, EggNOG, and GO databases. Species annotation was conducted using Kaiju to generate taxonomic abundance tables across six hierarchical levels (domain to species). High-quality reads were taxonomically classified via MMseqs2’s taxonomy module through sequence alignment against the NCBI nr database (v2021.10.11), applying the lowest common ancestor algorithm for precise species assignment. Functional annotation involved aligning protein sequences to KEGG, EggNOG, and GO databases using MMseqs2’s search module (sensitivity: 5.7). Taxonomic composition was analyzed with QIIME to produce abundance distribution tables, while results were visualized through MEGAN by mapping to the NCBI Taxonomy classification tree. Alpha diversity was quantified using Chao1, ACE, Shannon, and Simpson indices; beta diversity was assessed via principal coordinates analysis (PCoA). Linear discriminant analysis effect size (LEfSe)—integrating Kruskal–Wallis testing with linear discriminant analysis (LDA) effect size—identified significantly enriched taxa, with results visualized using R packages. All taxonomic abundance data were derived from relative abundance profiles; absolute microbial loads were not quantified in this study.

### Statistical analysis

Statistical analyses were conducted using GraphPad Prism software (GraphPad Software Inc., La Jolla, CA, United States). All values are reported as the mean ± standard error of the mean. Survival analysis was conducted using the Kaplan–Meier method, and differences between groups were assessed with the log-rank test. A one-way analysis of variance (ANOVA) was used for comparisons among multiple groups, while an unpaired *t*-test was applied for comparisons between two groups. For statistical analysis of the metagenomic sequencing data, inter-group differences were evaluated using permutational multivariate analysis of variance (Adonis) and analysis of similarities (ANOSIM). Species-level differential abundance between groups was determined using the metagenomeSeq algorithm, with Benjamini–Hochberg false discovery rate (FDR) correction applied; features with an adjusted *p*-value (adj. *p*) < 0.05 were considered statistically significant. A *p*-value of < 0.05 was considered statistically significant for all other analyses. Non-bacterial taxa (including eukaryotic and viral annotations) were filtered out prior to final abundance analysis. ^*^p < 0.05, ^**^p < 0.01, and ^***^p < 0.001.

## Results

### FLASH-RT demonstrates a protective effect on intestinal tissues

**Figure 2:**
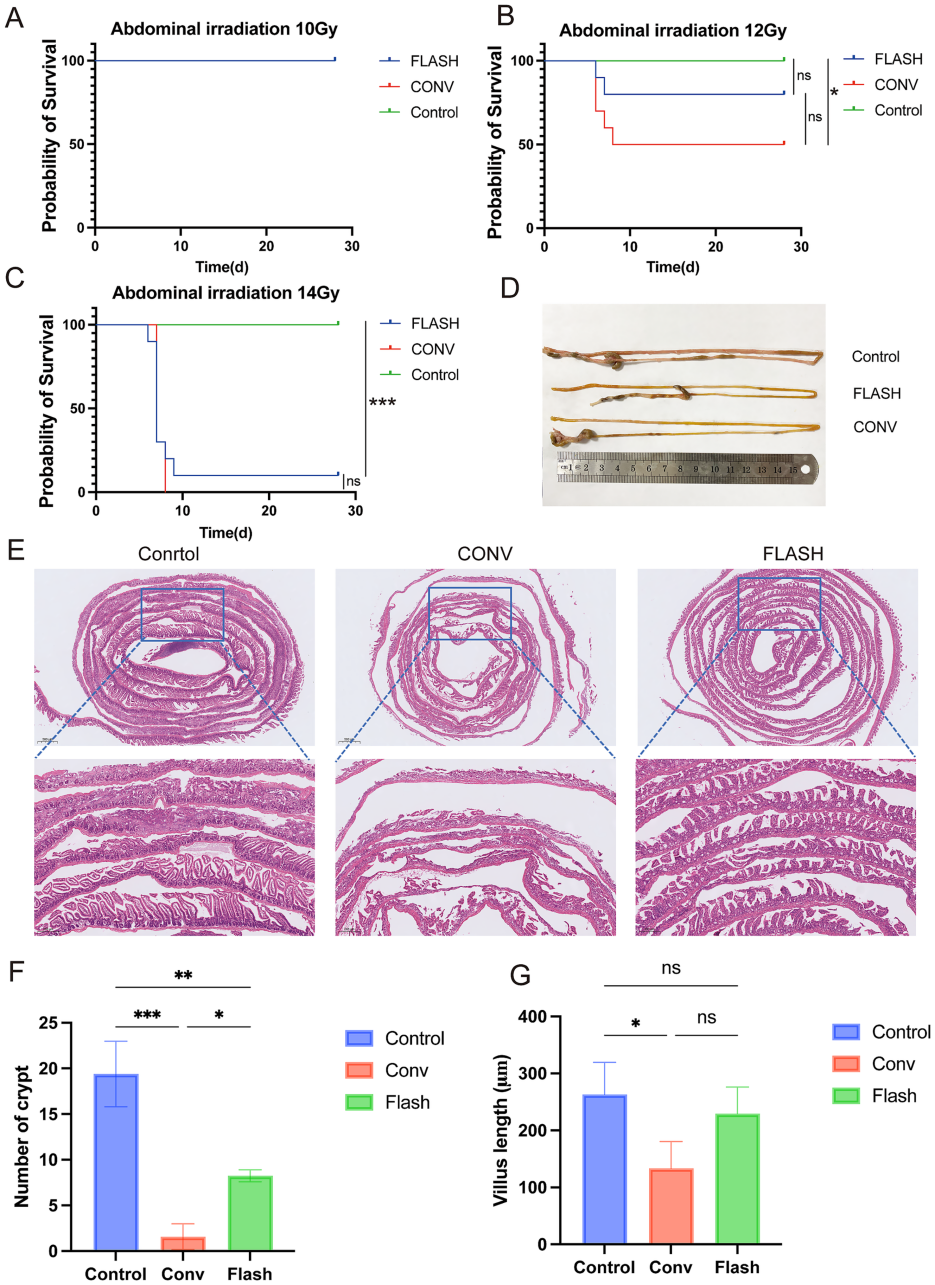
FLASH-RT alleviates damage to normal intestinal tissue. **(A–C)** Kaplan–Meier survival curves of healthy C57BL/6 mice following whole-abdomen irradiation at doses of 10 Gy, 12 Gy, and 14 Gy (*n* = 10 per group). **(D)** Representative *ex vivo* images of small intestinal tissue from each group after 12 Gy irradiation. **(E)** H&E-stained sections of small intestinal tissue from each group after 12 Gy irradiation. **(F)** Quantification of regenerated crypts per millimeter in each group after 12 Gy irradiation. **(G)** Comparison of villus height between groups after 12 Gy irradiation. ^*^p < 0.05, ^**^p < 0.01, and ^***^p < 0.001.

At 28 days post-whole-abdomen irradiation, no mortality was observed in any group of the 10 Gy dose group (Figure 2A). In the 12 Gy dose group, the survival rates for the control, FLASH, and CONV groups were 100, 80, and 50%, respectively. The survival rate of the control group was significantly higher than that of the CONV group (*p* < 0.05) and FLASH group, while the survival rate of the FLASH group was higher than that of the CONV group, though the difference was not statistically significant (*p* = 0.17, Figure 2B). In the 14 Gy dose group, the survival rates for the control, FLASH, and CONV groups were 100, 10, and 0%, respectively. The survival rate of the control group was significantly higher than that of the FLASH and CONV groups (*p* < 0.001). No significant difference in survival rates was observed between the FLASH and CONV groups (*p* > 0.05, Figure 2C). The specific survival numbers of mice in each group are shown in Supplementary Figure S3.

Histological examination of intestinal tissues with H&E staining in the 12 Gy group revealed that in the CONV group, there was extensive epithelial necrosis, detachment, and ulceration, with loss of intestinal villi and crypt structures, accompanied by significant inflammatory cell infiltration. In the FLASH group, epithelial necrosis and detachment were less severe, with mild inflammatory cell infiltration, partial villus atrophy, shortening, and crypt destruction. Overall, the extent of inflammation and damage was less pronounced in the FLASH group compared to the CONV group (Figures 2D,E). The number of intestinal crypts per mm in the control group was significantly higher than in the FLASH (*p* < 0.01) and CONV groups (*p* < 0.001). The FLASH group showed a significantly higher number of crypts per mm than the CONV group (*p* < 0.05, Figure 2F). The length of intestinal villi in the control group was significantly greater than in the FLASH and CONV groups (*p* < 0.05), while no significant difference in villus length was observed between the FLASH and CONV groups (*p* > 0.05, Figure 2G).

### Metagenomic analysis reveals the differences in gut microbiota and pathways among the groups

**Figure 3:**
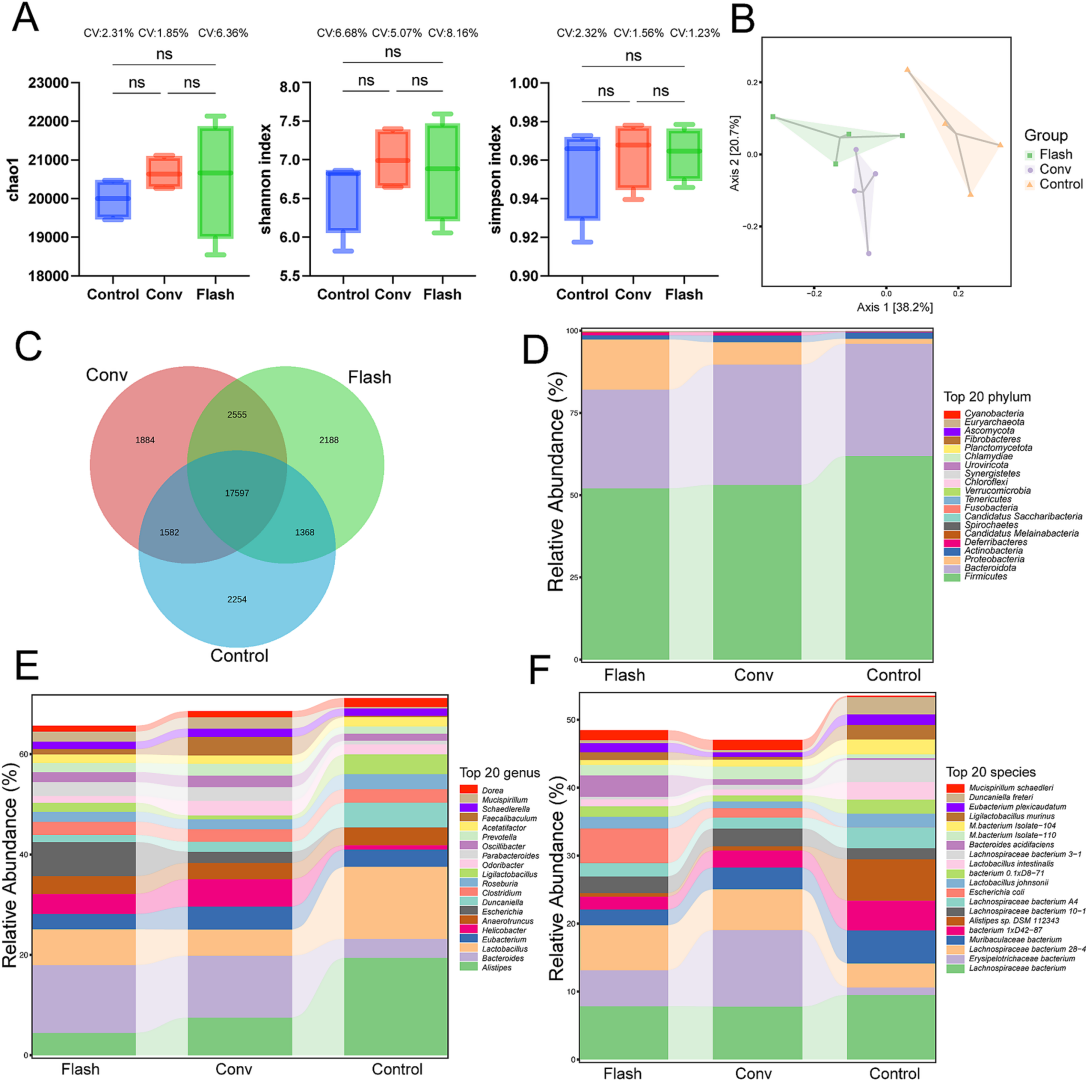
Preliminary comparison of the gut microbiome diversity across groups. **(A)** Alpha diversity was assessed using the Chao1, Simpson, and Shannon indices. The distributions are presented as boxplots, with the coefficient of variation (CV) indicated for each group. **(B)** Principal coordinate analysis (PCoA) of gut microbiota. **(C)** The Venn diagram illustrates the number of species shared and unique to each group. **(D)** Relative abundance of the top 20 bacteria at the phylum level. Top 20 phyla account for approximately 98.7% of the total microbiome abundance across all samples. **(E)** Relative abundance of the top 20 bacteria at the genus level. Top 20 genera account for approximately 89.2% of the total microbiome abundance across all samples. **(F)** Relative abundance of the top 20 bacteria at the species level. Top 20 species accounted for approximately 86.4% of the total microbiome across all samples.

The intestinal contents of normal mice from each group were collected 72 h after radiotherapy for metagenomic analysis. α-diversity analysis revealed no significant differences in microbial richness and diversity among the CONV, FLASH, and control groups. However, the coefficient of variation (CV) indicated greater intra-group variability in the FLASH group (Figure 3A). This observation was further supported by the clustered heatmap and NMDS ordination plots (Supplementary Figures S1, S2). PCoA analysis showed distinct separation of the microbial community structure among the three groups, with significant differences in microbial composition (Figure 3B). Based on the compositional profiles at the species level, the Venn diagram visually illustrates the number of species shared and unique to each group (Figure 3C). To explore whether the large number of differentially detected species included biologically meaningful taxa, we assessed the relative abundance of the top 200 unique species across groups. The majority of these accounted for less than 0.01% of the total abundance, suggesting that rare taxa detection due to reduced diversity post-irradiation may inflate the total species count. These data provide insights into the structural changes in the gut microbiome following different radiotherapy regimens after host-sequence removal. As shown in Figure 3D, at the phylum level, the microbiomes of all groups were dominated by Bacteroidota, Firmicutes, and Proteobacteria, together accounting for more than 95% of the total abundance. Compared with the control group, the FLASH group exhibited a modest increase in Proteobacteria and a decrease in Firmicutes, whereas the CONV group showed an increase in Bacteroidota with a reduction in Firmicutes. At the genus level (Figure 3E), the FLASH group displayed higher relative abundances of genera such as Bacteroides and Escherichia, but lower abundances of Lactobacillus compared to controls. In the CONV group, Bacteroides was also elevated, while genera such as Alistipes and Lactobacillus were reduced relative to the control group. Notably, Alistipes, a genus previously reported to be sensitive to radiation, was relatively preserved in the FLASH group compared with the CONV group. At the species level (Figure 3F), the FLASH group showed enrichment of *Bacteroides acidifaciens* and *Escherichia coli*, and a relative decrease in several Muribaculaceae taxa compared with controls. The CONV group, in contrast, exhibited increased relative abundances of certain Lachnospiraceae species and decreased levels of Alistipes species. To explore potential group-specific dominant taxa, we further identified species that were both relatively abundant and enriched in a single treatment group. Several species met this criterion, highlighting microbial signatures unique to each radiotherapy modality.

**Figure 4:**
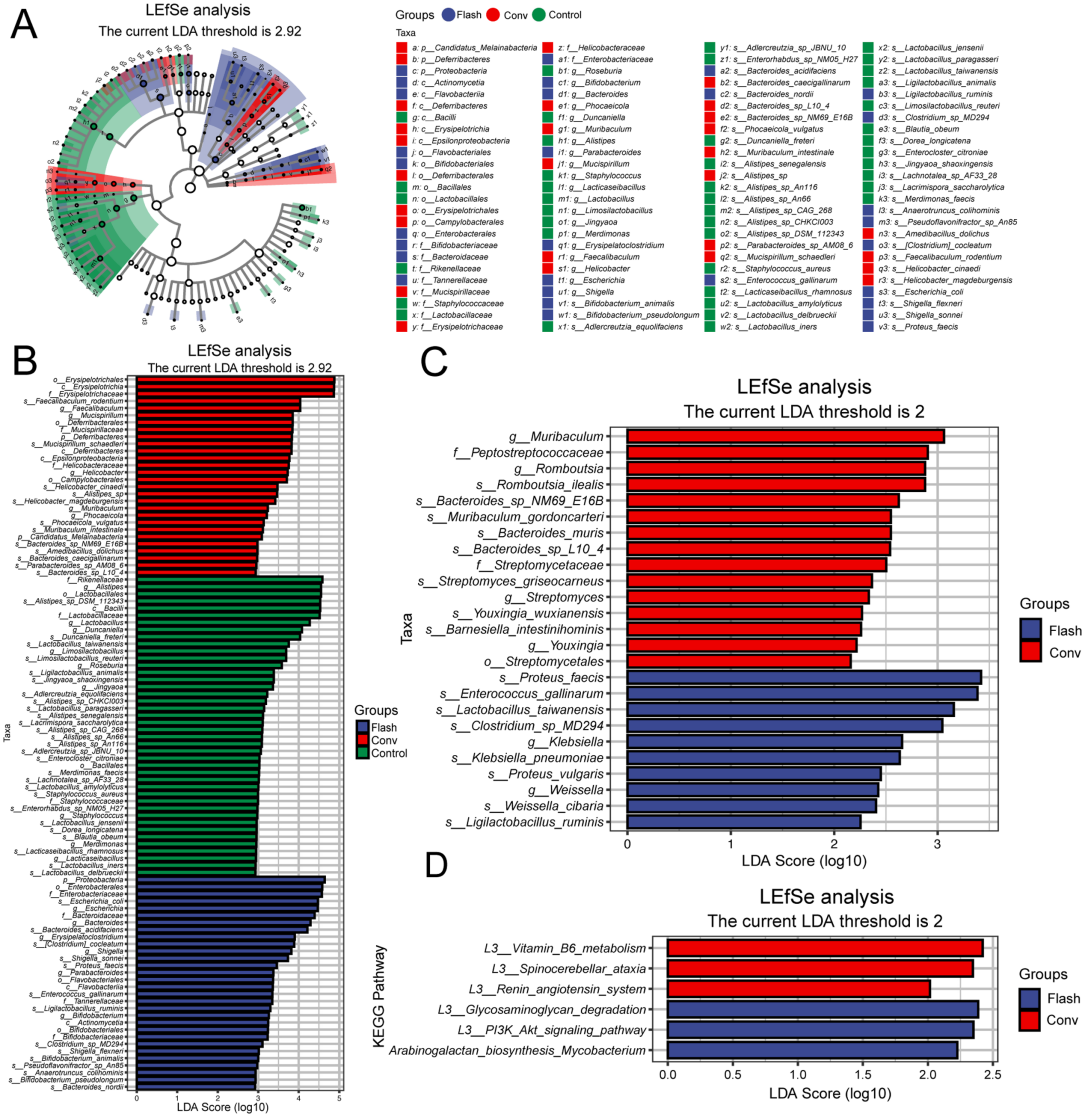
**(A)** Metagenomic analysis of gut microbiota and pathway differences between groups. The cladogram illustrates taxonomic hierarchies from phylum to genus (inner to outer circles). Node size reflects mean relative abundance, and node color indicates taxa with significant inter-group differences (LEfSe, Kruskal–Wallis test with FDR correction, LDA score >2.92. adj. *p* < 0.05). Color intensity corresponds to abundance magnitude. **(B)** Linear discriminant analysis (LDA) scores for differentially abundant taxa, highlighting enrichment patterns and biological effect size. Only features exceeding the significance threshold (LDA score >2.92; adj. *p* < 0.05) are displayed. **(C)** LEfSe-derived species bar plot showing significantly discriminant taxa between FLASH and CONV groups (LDA score >2, *p* < 0.05). **(D)** LEfSe functional bar plot displaying differentially enriched pathways (LDA score >2, *p* < 0.05). A color scale indicating the enrichment score (−log10 adjusted *p*-value) has been added to enhance interpretation.

Through differential species analysis (metagenomeSeq with Benjamini-Hochberg FDR correction), significant differences were observed at the species level between the FLASH group, the CONV group, and the control group (LDA score >2.92, adj. *p* < 0.05, Figures 4A,B). In Figure 4A, we present a heatmap of KEGG pathway enrichment based on metagenomic functional profiling. Several immune and metabolic pathways, such as NOD-like receptor signaling and amino acid biosynthesis, were differentially enriched across groups. FLASH-treated samples showed partial recovery of pathways related to epithelial barrier function and short-chain fatty acid metabolism. Subsequently, LEfSe was performed to identify the class-specific enrichment differences between the FLASH and CONV groups. Compared to the CONV group, the FLASH group exhibited significantly higher levels of *Weissella*, *Ligilactobacillus ruminis* and *Lactobacillus taiwanensis* (LDA score >2). These findings suggest that the observed differences in these microbial communities could be related to the alleviation of intestinal damage in the FLASH group (Figure 4C).

Functional prediction analysis through LEfSe further explored the differences in microbial functions between the FLASH and CONV groups. Pathway analysis revealed that, compared to the CONV group, FLASH significantly enhanced the functionality of pathways such as the glycosaminoglycan degradation, PI3K/Akt and arabinogalactan biosynthesis *Mycobacterium* signaling pathway (LDA score >2, Figure 4D). These results suggest that FLASH radiotherapy could alleviate damage to normal intestinal tissue by potentially modulating the distribution of intestinal microbiota and influencing several pathways.

## Discussion

This study tested three different doses to evaluate the protective effect of FLASH-RT on normal tissues. At a dose of 10 Gy, no mortality was observed in any group, while at 14 Gy, the entire conventional treatment group died, and only one mouse in the FLASH group survived. This suggests that the 14 Gy dose was too high, leading to near-total mortality, which was not conducive to observing the protective effects. Therefore, in subsequent experiments, we used a dose of 12 Gy, which resulted in approximately 50% mortality in the CONV group. Although no statistically significant difference in survival was found between the FLASH and CONV groups at the 12 Gy dose—likely due to the small sample size (*n* = 10)—the survival trends clearly indicate that FLASH-RT provides substantial protective effects. Further examination of the intestinal tissue from the 12 Gy group via HE staining revealed less tissue damage and fewer inflammatory cell infiltrations in the FLASH group. These findings further support the notion that high-energy X-ray FLASH-RT can confer protective effects on normal intestinal tissue.

LEfSe analysis revealed that several beneficial bacterial species in the FLASH group, including *Weissella*, *Ligilactobacillus ruminis* and *Lactobacillus taiwanensis*, were relatively enriched compared to the CONV group, based on their proportional abundance within the microbial community. Numerous studies have shown that *Weissella* produces antimicrobial exopolysaccharides, bacteriocins, hydrogen peroxide, and organic acids (Fusco et al., 2015), exhibiting antibacterial, antifungal, antioxidant, and anti-inflammatory properties, as well as enhancing the intestinal epithelial barrier function (Prado et al., 2020). The bacteriocins synthesized by *Weissella* demonstrate significant antimicrobial or bactericidal activity (Klaenhammer, 1993). The released organic acids also exert bactericidal effects against *Vibrio parahaemolyticus T.11* (Ahmed et al., 2022), while byproducts such as lactic acid and ethanol may enhance the anti-mycobacterial effect of lactic acid alone (Stedman et al., 2020). *Ligilactobacillus ruminis*, on the other hand, reduces the levels of pro-inflammatory cytokines such as IL-1β, TNF-*α*, and IL-17, thereby mitigating intestinal tissue damage. Additionally, *L. ruminis* increases the levels of short-chain fatty acids (SCFAs) in mouse feces, further modulating the gut microbiota balance and alleviating intestinal inflammation (Yang et al., 2021). Overall, *L. ruminis* exerts its beneficial effects by regulating inflammatory responses and restoring gut microbiota balance, thereby reducing inflammation and inhibiting intestinal tissue damage (Thorakkattu et al., 2022). These findings suggest that the intestinal protective effect of FLASH-RT may be associated with changes in the microbiome and its metabolites.

Interestingly, we observed that the genus Alistipes, which is known to exert protective effects against colitis and hepatic inflammation, was significantly depleted in the conventional irradiation group but relatively preserved following FLASH exposure. This finding aligns with prior studies indicating that Alistipes contributes to gut immune homeostasis, partly through production of short-chain fatty acids (SCFAs) and modulation of tryptophan metabolism. The preservation of Alistipes in FLASH-treated mice may thus underlie part of the observed reduction in intestinal injury, and warrants further mechanistic exploration in future studies.

LEfSe pathway analysis indicates that the protective effect of FLASH-RT on normal intestinal tissues might be related to several signaling pathways. Glycosaminoglycans (GAGs) are essential extracellular matrix components that play pivotal roles in intestinal barrier integrity and tissue repair (Tang et al., 2018). Studies indicate that GAG degradation exacerbates murine colitis symptoms, whereas inhibiting this process attenuates inflammatory responses (Belmiro et al., 2005). Specifically, probiotic lactobacilli that suppress GAG degradation ameliorate colitis by inhibiting both pro-inflammatory cytokine expression and bacterial GAG catabolism (Lee et al., 2009). This finding appears to contradict the protective effects of FLASH radiotherapy. However, another study suggests that reactive oxygen species (ROS) play a significant role in GAG degradation. ROS, with their high reactivity, can engage in multiple chemical reactions with GAGs, particularly hydroxyl radicals (•OH), which can break the glycosidic bonds in GAGs, thereby reducing their molecular weight and leading to moderate GAG degradation (Fuchs and Schiller, 2014). The consumption of ROS may mitigate oxidative stress damage to normal cells, creating favorable conditions for tissue repair and regeneration. While existing evidence shows a link between ROS and GAG degradation, further research is needed to better understand the specific role and extent of this pathway in the protective mechanism of FLASH radiotherapy.

The PI3K/Akt signaling pathway orchestrates critical cellular processes including proliferation, survival, and tissue repair. In inflammatory bowel disease, upregulation of the NRG1/ERBB_3_ axis activates PI3K/Akt signaling, sustaining intestinal epithelial proliferation and damage repair (Qiu et al., 2024). Studies have shown that activation of the PI3K/Akt/mTOR pathway is a key mechanism underlying radiation resistance in tumor cells across multiple radiotherapy models (Su et al., 2022). Activated Akt exerts its effects by stimulating the downstream mTOR pathway or by inhibiting proteins such as Bad and Caspase-9, which regulate cell proliferation, differentiation, apoptosis, and migration, contributing to radiation resistance in tumor cells (Chang et al., 2014; Chang et al., 2015; Horn et al., 2015). Based on these findings, we hypothesize that FLASH radiotherapy may enhance intestinal radioprotection by activating PI3K/Akt signaling in normal enterocytes, thereby promoting epithelial regeneration and repair.

Arabinogalactan (AG), a soluble dietary fiber with an excellent safety profile, has demonstrated immunomodulatory (Hamed et al., 2022), anticancer (Gong et al., 2020), anti-inflammatory, and antioxidant properties (Zheng et al., 2023). It also alleviates cisplatin-induced intestinal damage. Mechanistically, AG mitigates lipopolysaccharide induced damage to the small intestinal epithelial barrier by reducing inflammation and oxidative stress through modulation of the AMPK/SIRT1/NF-κB signaling pathway. In dry eye disease models, AG significantly reduced uric acid levels by 27% and reactive oxygen species (ROS) by 38%, thereby attenuating oxidative stress (Silvani et al., 2020). In acute liver injury models, AG treatment not only elevated levels of antioxidant enzymes, non-enzymatic antioxidants, and total hepatic antioxidant capacity but also significantly decreased lipid peroxidation in liver tissue (Sun et al., 2018). The enrichment of the arabinogalactan biosynthesis *Mycobacterium* signaling pathway in the flash group suggests that the protective effect of FLASH-RT on intestinal tissue may be related to the anti-inflammatory and antioxidant properties of arabinogalactan. These findings suggest that FLASH-RT may influence immune and stress-response pathways, including glycosaminoglycan degradation, PI3K/Akt and arabinogalactan biosynthesis *Mycobacterium* signaling, although the activation of these pathways could also reflect microbial stress or host-pathogen interaction rather than beneficial effects.

The high number of differentially detected species between irradiated and non-irradiated groups, as shown in Figure 3C, may initially appear surprising given the controlled housing conditions. However, this is likely attributable to deep sequencing sensitivity and the emergence of rare taxa after dominant species were depleted. Most of these species were detected at extremely low abundance, and their biological role remains uncertain. It is possible that microbial niche space became available following radiation-induced community collapse, allowing transient or low-abundance microbes to expand. Although FLASH-irradiated mice exhibited a microbiome profile that differed from that of conventionally irradiated animals, notable deviations from the control group remained. Alpha diversity in the FLASH group was partially restored compared to the CONV group, but remained lower than that of the unirradiated controls. NMDS and heatmap analyses also revealed that FLASH samples clustered closer to the CONV group than to the control group, indicating incomplete recovery. These findings suggest that FLASH-RT mitigates but does not fully reverse radiation-induced alterations in the gut microbiota.

Although metagenomic data preliminarily suggest that the protective effect of FLASH-RT on normal intestinal tissue may be linked to the modulation of gut microbiota composition, several limitations exist in this study. The diversity analysis results show substantial within-group variation in the FLASH group, and this high heterogeneity may impact the statistical significance of specific microbial changes, thereby interfering with the stability of the conclusions. However, due to the limited number of experimental animal samples, excluding samples that deviate from the norm may introduce substantial selection bias. Furthermore, inherent differences in the microbiota state across different irradiation batches of mice complicate sample addition, potentially increasing systemic bias and affecting the reliability and comparability of the results. In this experiment, 80% of the mice survived after receiving 12 Gy FLASH-RT, while 20% died, suggesting individual variability in the protective effect of FLASH. Combined with the observed heterogeneity in the microbiota species of the FLASH group, we hypothesize that the heterogeneity within the FLASH group may be related to differences in individual microbiota recovery abilities, which could be a key factor in the protective effect of FLASH. Therefore, excluding individuals with large differences might obscure the biological significance of individual microbiota recovery processes. To more accurately reflect the individual variability after FLASH-RT, we retained all raw data and approached data analysis with caution, avoiding overinterpretation of potential causal relationships. Through this approach, we believe that this study provides preliminary insights into the changes in gut microbiota following FLASH-RT intervention and offers new directions for exploring the mechanisms behind individual responses to FLASH-RT.

In summary, the protective effects of FLASH-RT on the intestine may be associated with multiple microbiota and signaling pathways. Gut microbiota may exert their effects through specific metabolites, such as short-chain fatty acids and polysaccharides. However Liberles et al. (2013) proposed that confirming causal mechanisms requires further experimental validation. This study is based on metagenomic analyses and does not include targeted metabolomics; therefore, functional inferences derived solely from metagenomic pathway annotations should be interpreted with caution. Causal relationships between microbial community changes and host effects require experimental validation. Future research should further explore the specific roles and mechanisms of different microbiota in this process to better understand the potential of FLASH-RT in intestinal protection.

## Conclusion

Compared to CONV-RT, high-energy X-ray FLASH irradiation exerts radioprotective effects on normal intestinal tissue. Alterations in the gut microbiota and associated signaling pathways may be linked to the protective effects of FLASH.

## Data Availability

The datasets presented in this study are publicly available. This data can be found here: https://ngdc.cncb.ac.cn/gsa, accession number CRA028854.

## References

[ref1] Ahmed S. Singh S. Singh V. Roberts K. D. Zaidi A. Rodriguez-Palacios A. (2022). The *Weissella* genus: clinically treatable bacteria with antimicrobial/probiotic effects on inflammation and cancer. Microorganisms 10:2427. doi: 10.3390/microorganisms10122427, PMID: 36557680 PMC9788376

[ref2] Bao Z. Wang D. Chen S. Chen M. Jiang D. Yang C.. (2019). Optimal dose limitation strategy for bone marrow sparing in intensity-modulated radiotherapy of cervical cancer. Radiat. Oncol. 14:118. doi: 10.1186/s13014-019-1324-y, PMID: 31378200 PMC6681496

[ref3] Baumann M. Krause M. Overgaard J. Debus J. Bentzen S. M. Daartz J.. (2016). Radiation oncology in the era of precision medicine. Nat. Rev. Cancer 16, 234–249. doi: 10.1038/nrc.2016.18, PMID: 27009394

[ref4] Belmiro C. L. Souza H. S. Elia C. C. Castelo-Branco M. T. Silva F. R. Machado R. L.. (2005). Biochemical and immunohistochemical analysis of glycosaminoglycans in inflamed and non-inflamed intestinal mucosa of patients with Crohn’s disease. Int. J. Color. Dis. 20, 295–304. doi: 10.1007/s00384-004-0677-2, PMID: 15660268

[ref5] Bourhis J. Sozzi W. J. Jorge P. G. Gaide O. Bailat C. Duclos F.. (2019). Treatment of a first patient with FLASH-radiotherapy. Radiother. Oncol. 139, 18–22. doi: 10.1016/j.radonc.2019.06.019, PMID: 31303340

[ref6] Cesar J. P. Abouzahr F. Crespo P. Gajda M. Kuo A. Mawlawi O.. (2024). First PET studies of a FLASH proton beam: summary and future prospects. Bio-Algorithms Med-Syst. 20, 49–54. doi: 10.5604/01.3001.0054.9140

[ref7] Chang L. Graham P. H. Hao J. Ni J. Bucci J. Cozzi P. J.. (2014). PI3K/Akt/mTOR pathway inhibitors enhance radiosensitivity in radioresistant prostate cancer cells through inducing apoptosis, reducing autophagy, suppressing NHEJ and HR repair pathways. Cell Death Dis. 5:e1437. doi: 10.1038/cddis.2014.415, PMID: 25275598 PMC4237243

[ref8] Chang L. Graham P. H. Ni J. Hao J. Bucci J. Cozzi P. J.. (2015). Targeting PI3K/Akt/mTOR signaling pathway in the treatment of prostate cancer radioresistance. Crit. Rev. Oncol. Hematol. 96, 507–517. doi: 10.1016/j.critrevonc.2015.07.005, PMID: 26253360

[ref10] Chow J. C. L. Ruda H. E. (2024). Mechanisms of action in FLASH radiotherapy: a comprehensive review of physicochemical and biological processes on cancerous and normal cells. Cells 13:835. doi: 10.3390/cells13100835, PMID: 38786057 PMC11120005

[ref12] Durak-Kozica M. S. E. Swakoń J. Moskal P. (2023). Application of an ultra-high dose rate (FLASH) proton beam for the 3D cancer cell model—a proof of concept. Bio-Algorithms Med-Syst. 19, 31–34. doi: 10.5604/01.3001.0054.1820

[ref13] Eling L. Bouchet A. Nemoz C. Djonov V. Balosso J. Laissue J.. (2019). Ultra high dose rate synchrotron microbeam radiation therapy. Preclinical evidence in view of a clinical transfer. Radiother. Oncol. 139, 56–61. doi: 10.1016/j.radonc.2019.06.030, PMID: 31307824

[ref14] Favaudon V. Caplier L. Monceau V. Pouzoulet F. Sayarath M. Fouillade C.. (2014). Ultrahigh dose-rate FLASH irradiation increases the differential response between normal and tumor tissue in mice. Sci. Transl. Med. 6:245ra93. doi: 10.1126/scitranslmed.3008973, PMID: 25031268

[ref15] Fuchs B. Schiller J. (2014). Glycosaminoglycan degradation by selected reactive oxygen species. Antioxid. Redox Signal. 21, 1044–1062. doi: 10.1089/ars.2013.5634, PMID: 24125575

[ref16] Fusco V. Quero G. M. Cho G. S. Kabisch J. Meske D. Neve H.. (2015). The genus *Weissella*: taxonomy, ecology and biotechnological potential. Front. Microbiol. 6:155. doi: 10.3389/fmicb.2015.00155, PMID: 25852652 PMC4362408

[ref17] Gao F. Yang Y. Zhu H. Wang J. Xiao D. Zhou Z.. (2022). First demonstration of the FLASH effect with ultrahigh dose rate high-energy X-rays. Radiother. Oncol. 166, 44–50. doi: 10.1016/j.radonc.2021.11.004, PMID: 34774651

[ref18] Gong G. Liu Q. Deng Y. Dang T. Dai W. Liu T.. (2020). Arabinogalactan derived from *Lycium barbarum* fruit inhibits cancer cell growth via cell cycle arrest and apoptosis. Int. J. Biol. Macromol. 149, 639–650. doi: 10.1016/j.ijbiomac.2020.01.251, PMID: 31991207

[ref19] Groselj B. Ruan J. L. Scott H. Gorrill J. Nicholson J. Kelly J.. (2018). Radiosensitization *in vivo* by histone deacetylase inhibition with no increase in early normal tissue radiation toxicity. Mol. Cancer Ther. 17, 381–392. doi: 10.1158/1535-7163.Mct-17-0011, PMID: 28839000 PMC5712223

[ref20] Hageman E. Che P. P. Dahele M. Slotman B. J. Sminia P. (2022). Radiobiological aspects of FLASH radiotherapy. Biomolecules 12:1376. doi: 10.3390/biom12101376, PMID: 36291585 PMC9599153

[ref21] Hamed M. Coelho E. Bastos R. Evtuguin D. V. Ferreira S. S. Lima T.. (2022). Isolation and identification of an arabinogalactan extracted from pistachio external hull: assessment of immunostimulatory activity. Food Chem. 373:131416. doi: 10.1016/j.foodchem.2021.131416, PMID: 34717082

[ref22] Horn D. Hess J. Freier K. Hoffmann J. Freudlsperger C. (2015). Targeting EGFR-PI3K-AKT-mTOR signaling enhances radiosensitivity in head and neck squamous cell carcinoma. Expert Opin. Ther. Targets 19, 795–805. doi: 10.1517/14728222.2015.1012157, PMID: 25652792

[ref23] Hughes J. R. Parsons J. L. (2020). FLASH radiotherapy: current knowledge and future insights using proton-beam therapy. Int. J. Mol. Sci. 21:6492. doi: 10.3390/ijms21186492, PMID: 32899466 PMC7556020

[ref24] Klaenhammer T. R. (1993). Genetics of bacteriocins produced by lactic acid bacteria. FEMS Microbiol. Rev. 12, 39–85. doi: 10.1111/j.1574-6976.1993.tb00012.x, PMID: 8398217

[ref25] Lang K. (2024). Image-guided FLASH proton therapy. A dream? Naivety? Arrogance? Or a necessity? Bio-Algorithms Med-Syst. 20, 17–26. doi: 10.5604/01.3001.0054.8930

[ref26] Lee B. Lee J. H. Lee H. S. Bae E. A. Huh C. S. Ahn Y. T.. (2009). Glycosaminoglycan degradation-inhibitory lactic acid bacteria ameliorate 2,4,6-trinitrobenzenesulfonic acid-induced colitis in mice. J. Microbiol. Biotechnol. 19, 616–621. doi: 10.4014/jmb.0808.479, PMID: 19597321

[ref27] Levy K. Natarajan S. Wang J. Chow S. Eggold J. T. Loo P. E.. (2020). Abdominal flash irradiation reduces radiation-induced gastrointestinal toxicity for the treatment of ovarian cancer in mice. Sci. Rep. 10:21600. doi: 10.1038/s41598-020-78017-7 33303827 PMC7728763

[ref28] Liberles D. A. Teufel A. I. Liu L. Stadler T. (2013). On the need for mechanistic models in computational genomics and metagenomics. Genome Biol. Evol. 5, 2008–2018. doi: 10.1093/gbe/evt151, PMID: 24115604 PMC3814209

[ref29] Lu Q. Liang Y. Tian S. Jin J. Zhao Y. Fan H. (2023). Radiation-induced intestinal injury: injury mechanism and potential treatment strategies. Toxics 11:1011. doi: 10.3390/toxics11121011, PMID: 38133412 PMC10747544

[ref30] Ma Y. Zhang W. Zhao Z. Lv J. Chen J. Yan X.. (2024). Current views on mechanisms of the FLASH effect in cancer radiotherapy. Natl. Sci. Rev. 11:nwae350. doi: 10.1093/nsr/nwae350, PMID: 39479528 PMC11523052

[ref31] Melia E. Parsons J. L. (2023). DNA damage and repair dependencies of ionising radiation modalities. Biosci. Rep. 43:BSR20222586. doi: 10.1042/bsr20222586, PMID: 37695845 PMC10548165

[ref32] Montay-Gruel P. Acharya M. M. Petersson K. Alikhani L. Yakkala C. Allen B. D.. (2019). Long-term neurocognitive benefits of FLASH radiotherapy driven by reduced reactive oxygen species. Proc. Natl. Acad. Sci. U.S.A. 116, 10943–10951. doi: 10.1073/pnas.1901777116, PMID: 31097580 PMC6561167

[ref33] Montay-Gruel P. Bouchet A. Jaccard M. Patin D. Serduc R. Aim W.. (2018). X-rays can trigger the FLASH effect: ultra-high dose-rate synchrotron light source prevents normal brain injury after whole brain irradiation in mice. Radiother. Oncol. 129, 582–588. doi: 10.1016/j.radonc.2018.08.016, PMID: 30177374

[ref34] Montay-Gruel P. Corde S. Laissue J. A. Bazalova-Carter M. (2022). FLASH radiotherapy with photon beams. Med. Phys. 49, 2055–2067. doi: 10.1002/mp.15222, PMID: 34519042

[ref35] Nath R. Epp E. R. Laughlin J. S. Swanson W. P. Bond V. P. (1984). Neutrons from high-energy X-ray medical accelerators: an estimate of risk to the radiotherapy patient. Med. Phys. 11, 231–241. doi: 10.1118/1.595497, PMID: 6429495

[ref36] Overgaard J. Bartelink H. (1995). About tolerance and quality. An important notice to all radiation oncologists. Radiother. Oncol. 35, 1–3. doi: 10.1016/0167-8140(95)01568-2, PMID: 7569010

[ref37] Prado G. K. S. Torrinha K. C. Cruz R. E. Gonçalves A. B. B. Silva C. A. V. Oliveira F. M. S.. (2020). *Weissella paramesenteroides* WpK4 ameliorate the experimental amoebic colitis by increasing the expression of MUC-2 and the intestinal epithelial regeneration. J. Appl. Microbiol. 129, 1706–1719. doi: 10.1111/jam.14671, PMID: 32320114

[ref38] Qiu D. Xu S. Ji K. Tang C. (2024). Myeloid cell-derived IL-1 signaling damps neuregulin-1 from fibroblasts to suppress colitis-induced early repair of the intestinal epithelium. Int. J. Mol. Sci. 25:4469. doi: 10.3390/ijms25084469, PMID: 38674054 PMC11050633

[ref39] Rahman M. Trigilio A. Franciosini G. Moeckli R. Zhang R. Böhlen T. T. (2022). FLASH radiotherapy treatment planning and models for electron beams. Radiother. Oncol. 175, 210–221. doi: 10.1016/j.radonc.2022.08.009, PMID: 35964763

[ref40] Ruan J. L. Lee C. Wouters S. Tullis I. D. C. Verslegers M. Mysara M.. (2021). Irradiation at ultra-high (FLASH) dose rates reduces acute normal tissue toxicity in the mouse gastrointestinal system. Int. J. Radiat. Oncol. Biol. Phys. 111, 1250–1261. doi: 10.1016/j.ijrobp.2021.08.004, PMID: 34400268 PMC7612009

[ref41] Ryoo S. B. Kim J. S. Kim M. S. Kim K. Yu S. A. Bae M. J.. (2016). High-dose radiation-induced changes in murine small intestinal motility: are the changes in the interstitial cells of Cajal or in the enteric nervous system? Radiat. Res. 185, 39–49. doi: 10.1667/rr14132.1, PMID: 26720798

[ref42] Schaue D. McBride W. H. (2015). Opportunities and challenges of radiotherapy for treating cancer. Nat. Rev. Clin. Oncol. 12, 527–540. doi: 10.1038/nrclinonc.2015.120, PMID: 26122185 PMC8396062

[ref43] Shan L. Zhou Z. Yiwei Y. Liu Y. Wang J. Zhang D.. (2023). 10 MeV, >80 Gy/s@1 m photon FLASH radiotherapy source. High Power Laser Part. Beams 35, 114–115. doi: 10.11884/HPLPB202335.230412

[ref44] Shiraishi Y. Matsuya Y. Fukunaga H. (2024). Possible mechanisms and simulation modeling of FLASH radiotherapy. Radiol. Phys. Technol. 17, 11–23. doi: 10.1007/s12194-023-00770-x, PMID: 38184508

[ref45] Silvani L. Bedei A. De Grazia G. Remiddi S. (2020). Arabinogalactan and hyaluronic acid in ophthalmic solution: experimental effect on xanthine oxidoreductase complex as key player in ocular inflammation (*in vitro* study). Exp. Eye Res. 196:108058. doi: 10.1016/j.exer.2020.108058, PMID: 32380019

[ref46] Simmons D. A. Lartey F. M. Schüler E. Rafat M. King G. Kim A.. (2019). Reduced cognitive deficits after FLASH irradiation of whole mouse brain are associated with less hippocampal dendritic spine loss and neuroinflammation. Radiother. Oncol. 139, 4–10. doi: 10.1016/j.radonc.2019.06.006, PMID: 31253467

[ref47] Srinivasan D. Subbarayan R. Srivastava N. Radhakrishnan A. Adtani P. N. Chauhan A.. (2024). A comprehensive overview of radiation therapy impacts of various cancer treatments and pivotal role in the immune system. Cell Biochem. Funct. 42:e4103. doi: 10.1002/cbf.4103, PMID: 39073207

[ref48] Stedman A. van Vliet A. H. M. M A. C. Gutierrez-Merino J. (2020). Gut commensal bacteria show beneficial properties as wildlife probiotics. Ann. N. Y. Acad. Sci. 1467, 112–132. doi: 10.1111/nyas.14302 32026493

[ref49] Su Y. C. Lee W. C. Wang C. C. Yeh S. A. Chen W. H. Chen P. J. (2022). Targeting PI3K/AKT/mTOR signaling pathway as a radiosensitization in head and neck squamous cell carcinomas. Int. J. Mol. Sci. 23:15749. doi: 10.3390/ijms232415749, PMID: 36555391 PMC9778923

[ref50] Sun J. Wen X. Liu J. Kan J. Qian C. Wu C.. (2018). Protective effect of an arabinogalactan from black soybean against carbon tetrachloride-induced acute liver injury in mice. Int. J. Biol. Macromol. 117, 659–664. doi: 10.1016/j.ijbiomac.2018.05.203, PMID: 29852225

[ref51] Tang F. Lord M. S. Stallcup W. B. Whitelock J. M. (2018). Cell surface chondroitin sulphate proteoglycan 4 (CSPG4) binds to the basement membrane heparan sulphate proteoglycan, perlecan, and is involved in cell adhesion. J. Biochem. 163, 399–412. doi: 10.1093/jb/mvy008, PMID: 29462330 PMC5905647

[ref52] Thorakkattu P. Khanashyam A. C. Shah K. Babu K. S. Mundanat A. S. Deliephan A.. (2022). Postbiotics: current trends in food and pharmaceutical industry. Foods 11:3094. doi: 10.3390/foods11193094, PMID: 36230169 PMC9564201

[ref53] Vozenin M. C. De Fornel P. Petersson K. Favaudon V. Jaccard M. Germond J. F.. (2019). The advantage of FLASH radiotherapy confirmed in mini-pig and cat-cancer patients. Clin. Cancer Res. 25, 35–42. doi: 10.1158/1078-0432.Ccr-17-3375, PMID: 29875213

[ref54] Yan O. Wang S. Wang Q. Wang X. (2024). FLASH radiotherapy: mechanisms of biological effects and the therapeutic potential in cancer. Biomolecules 14:754. doi: 10.3390/biom14070754, PMID: 39062469 PMC11275005

[ref55] Yang B. Li M. Wang S. Ross R. P. Stanton C. Zhao J.. (2021). *Lactobacillus ruminis* alleviates DSS-induced colitis by inflammatory cytokines and gut microbiota modulation. Foods 10:1349. doi: 10.3390/foods10061349, PMID: 34208038 PMC8230674

[ref56] Zheng J. Gong S. Han J. (2023). Arabinogalactan alleviates lipopolysaccharide-induced intestinal epithelial barrier damage through adenosine monophosphate-activated protein kinase/silent information regulator 1/nuclear factor kappa-B signaling pathways in Caco-2 cells. Int. J. Mol. Sci. 24:5337. doi: 10.3390/ijms242015337, PMID: 37895018 PMC10607795

