# Supplementary material for: Exploring the role of intestinal microbiota in mitigating acute radiation-induced intestinal injury through high-energy X-ray FLASH radiotherapy via metagenomic analysis
Source: Front Microbiol. 2025 Oct 17;16:1601244. doi: 10.3389/fmicb.2025.1601244 (PMC12575193; doi:10.3389/fmicb.2025.1601244)
Supplement: Supplementary file 1 [file Supplementary_file_1.docx]

**Supplementary materials**

**Figure S1**


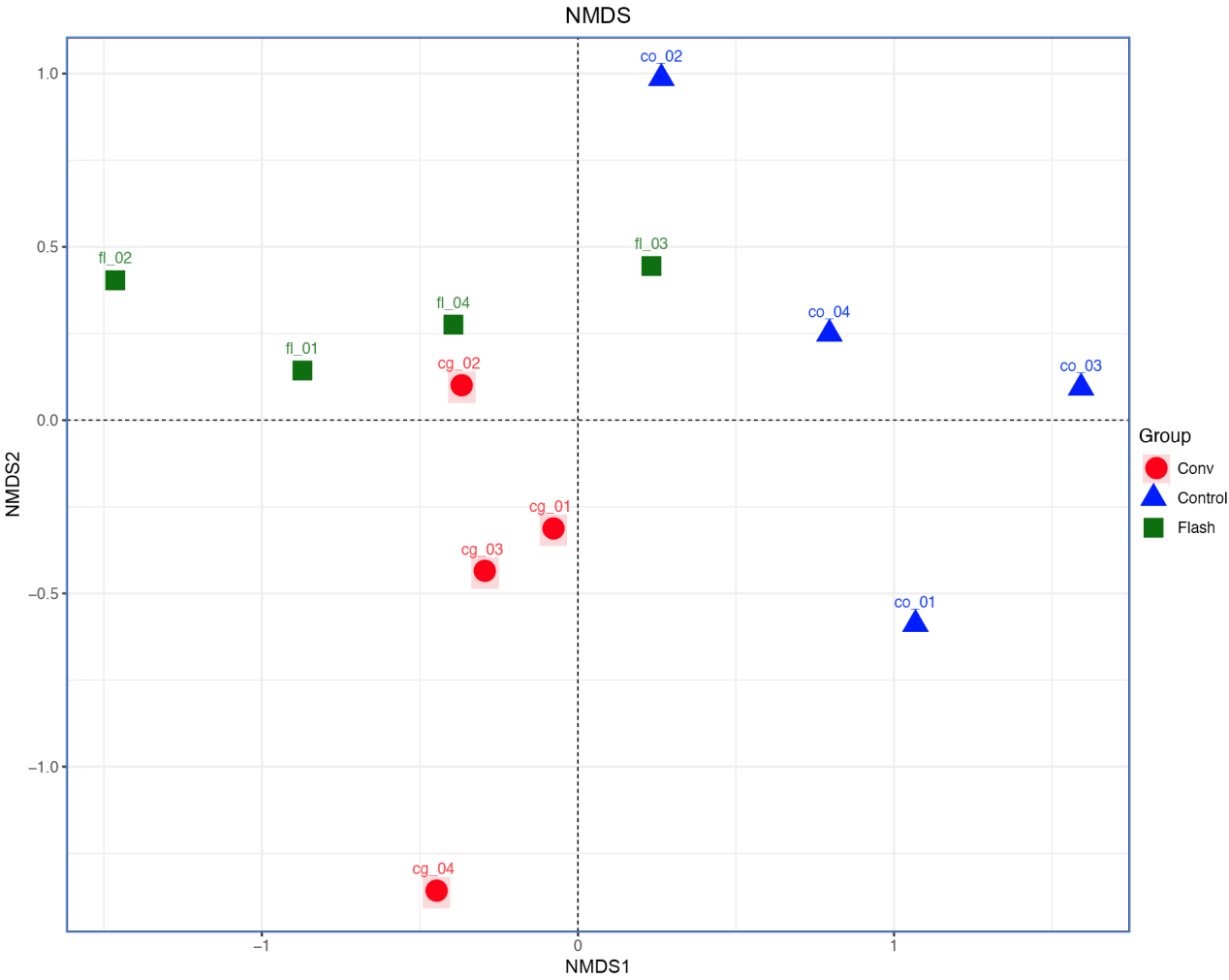


****Figure S1.**** NMDS ordination plot based on species composition. In the plot, each point represents a sample, with different colors indicating different experimental groups. The closer two points are, the smaller the dissimilarity in species composition between the corresponding samples, indicating a higher degree of similarity.

****Figure S2****


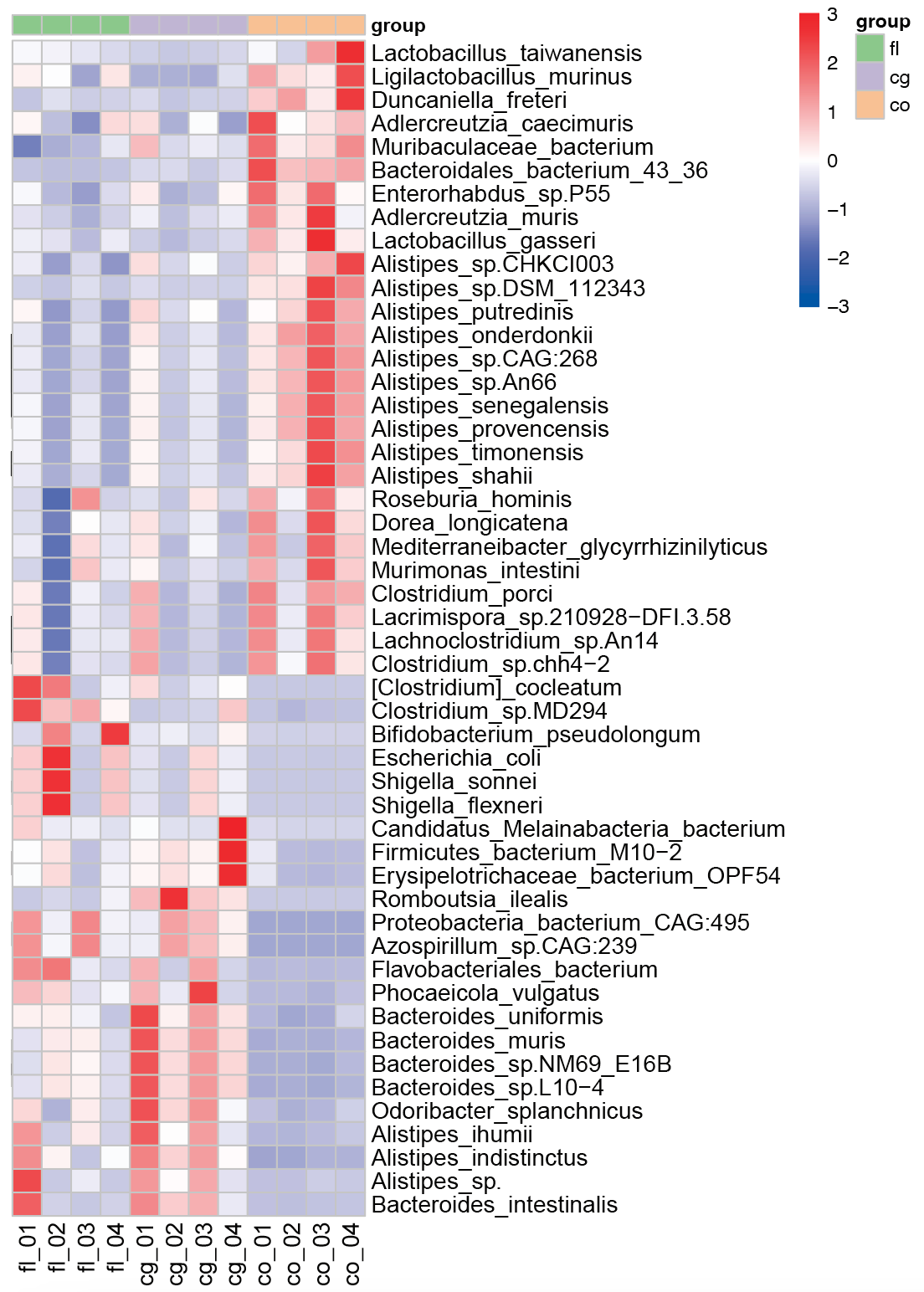


**Figure S2.** Clustered heatmap of the top 50 differential species at the species level. Red indicates species with relatively high abundance in the corresponding samples, while green represents species with relatively low abundance.

**Figure S3**


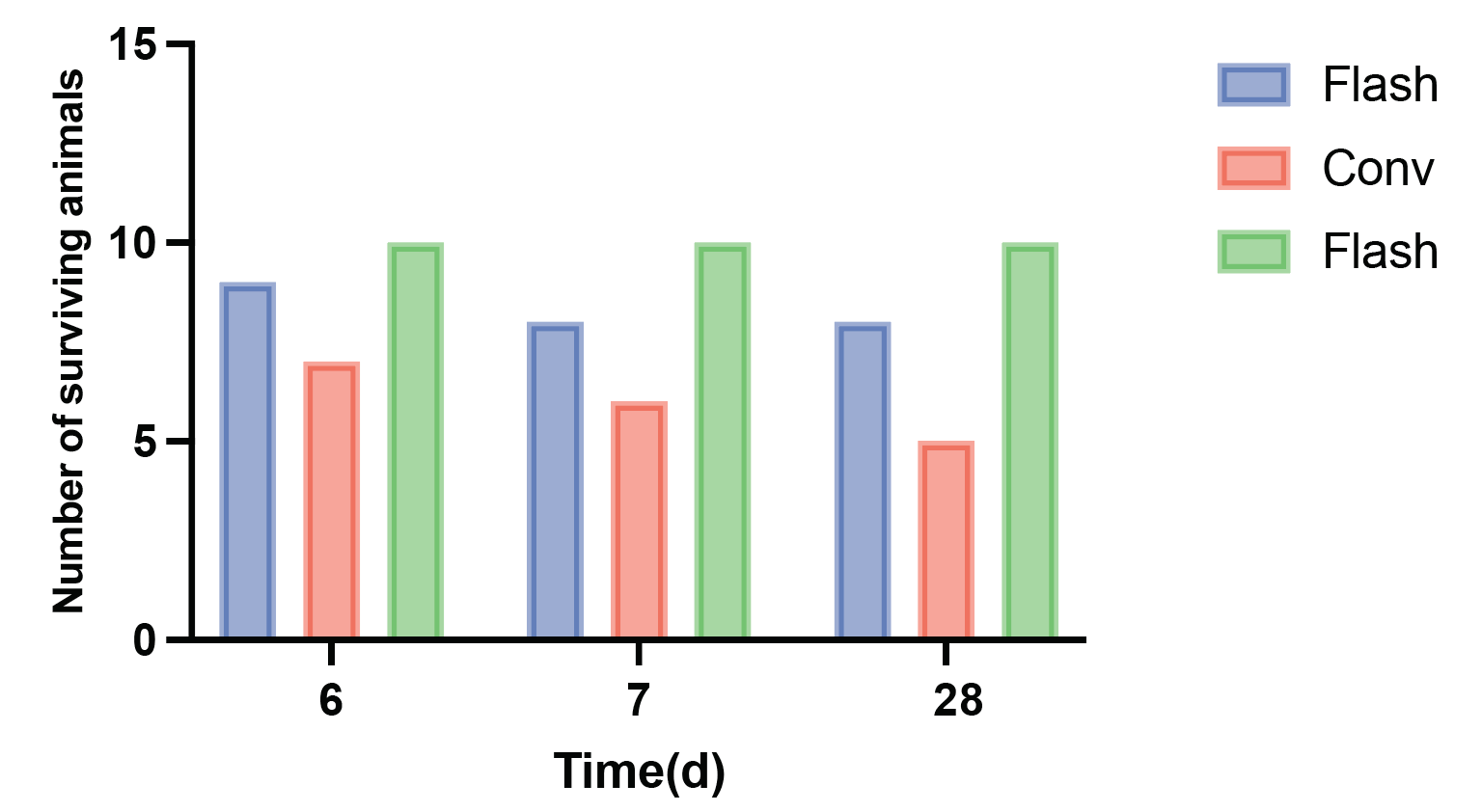


**Figure S3.** Bar charts of the number of surviving animals at different time points.Bar chart depicting the number of surviving animals at each time point for each group following 12 Gy whole-abdomen irradiation.
